# A Manual of Histology

**Published:** 1873-03

**Authors:** 


### Books Reviewed.

*A Manual of Histology.* By Prof. S. Stricker, in co-operation with Th. Meynert, F. Von Recklinghausen, Max Schultz, W. Waldeyer and others. Translated by Henry Power, of London; Jas. J. Putnam and J. Orne Green, of Boston; Henry C. Eno, Thomas E. Satterthwaite, Edward C. Seguin, Lucius D. Bulkley, Edward L. Keyes and Francis E. Delafield, of New York. Edited by ALBERT H. BUCK, Assistant Aural Surgeon to the New York Eye and Ear Infirmary. With 431 Illustrations. New York: Wm. Wood & Co., 1872. Buffalo: H. H. Otis.

The subject of Histology is daily attracting more and more attention from American minds, and what has for some time been one of the practical departments of European, and especially of German medical education, is, we are happy to see, being introduced into the medical schools of this country. The student of Histology has labored under many difficulties in the pursuit of his calling, one of the most embarrassing being the lack of a suitable text book or manual. He has had to content himself with what little information he could gain by hard labor from the more advanced treatises intended for practical workers in the science, and could only ascertain the state of information in regard to any particular branch by careful and tiresome research among the scattered monographs which had been published upon the subject. In many departments he was compelled to rely wholly upon himself, and was often led into mistaken conclusions, which may, in a measure, account for the diversity of opinions held on some subjects by histologists. The large and complete manual before us is published under the general supervision of Prof. Stricker, whose reputation as a microscopist is well known to every professional man. Assisted by some of the most noted observers of Europe, he has produced a manual in every way a credit to his name. With contributions from so many pens, the style of the work would, from a necessity, be diversified; this, however, adds rather than detracts from its value, as it enables the reader to observe and compare the different modes of observing facts adopted by the writers.

Of the English translation the first four hundred and six pages are the work of Mr. Henry Power, of London, the balance of the work being performed by American physicians. The subject matter is carefully arranged under the supervision of Dr. Albert H. Buck, of New York. The work is divided into thirty-eight chapters, to which have been added two supplemental articles. Of these, but two chapters and the introduction are from the pen of Prof. Stricker himself; the introduction treating of the general methods of investigation, the other chapters treating of the general character of Nerve Cells, (Chap. I.) and Development of Simple Tissues, (Chap. XXXVIII.) All the articles, however, have passed under the general supervision of Prof. Stricker, and may, as a general thing, be taken to represent the present state of knowledge concerning their several subjects. The illustrations are all that could be asked to explain the text, and are sufficiently numerous. The type is clear and the book is gotten up in a style which reflects credit upon authors, translators and publishers. No student of histology need now give up the study for want of a text-book, the manual of Stricker supplies all, or nearly all that he needs.

